# Gene-gene interactions in gastrointestinal cancer susceptibility

**DOI:** 10.18632/oncotarget.11701

**Published:** 2016-08-30

**Authors:** Jineun Kim, Seoyun Yum, Changwon Kang, Suk-Jo Kang

**Affiliations:** ^1^ Department of Biological Sciences, Korea Advanced Institute of Science and Technology, Daejeon, Korea

**Keywords:** colorectal cancer, epistasis, esophageal cancer, gastric cancer, gene-gene interaction

## Abstract

Cancer arises from complex, multi-layer interactions between diverse genetic and environmental factors. Genetic studies have identified multiple loci associated with tumor susceptibility. However, little is known about how germline polymorphisms interact with one another and with somatic mutations within a tumor to mediate acquisition of cancer traits. Here, we survey recent studies showing gene-gene interactions, also known as epistases, affecting genetic susceptibility in colorectal, gastric and esophageal cancers. We also catalog epistasis types and cancer hallmarks with respect to the interacting genes. A total of 22 gene variation pairs displayed all levels of statistical epistasis, including synergistic, redundant, suppressive and co-suppressive interactions. Five genes primarily involved in base excision repair formed a linear topology in the interaction network, *MUTYH*-*OGG1*-*XRCC1*-*PARP1*-*MMP2*, and three genes in mTOR cell-proliferation pathway formed another linear network, *PRKAG2*-*RPS6KB1*-*PIK3CA*. Discrete pairwise epistasis was also found in nucleotide excision repair, detoxification, proliferation, TP53, TGF-β and other pathways. We propose that three modes of biological interaction underlie the molecular mechanisms for statistical epistasis. The direct binding, linear pathway and convergence modes can exhibit any level of statistical epistasis in susceptibility to gastrointestinal cancers, and this is likely true for other complex diseases as well. This review highlights the link between cancer hallmarks and susceptibility genes.

## INTRODUCTION

Mutation of oncogenes and tumor suppressor genes is a primary force underlying oncogenesis and cancer progression. To identify genetic factors involved in tumor traits, most studies have focused on tumor-associated loci that are presumed to include gene(s) responsible for cancer development. This type of research reveals germline tumor susceptibility elements. However, because tumors acquire many malignant traits through somatic mutations, the success of such approaches depends on identifying genetic variations in known oncogenes and tumor suppressor genes, and such variations strongly influencing gene expression or function.

As is common for complex diseases, development of cancer traits occurs through the interaction of multiple genes and environmental factors. Additionally, tumors actively engage with nearby cells, such as stromal and immune cells recruited to the tumor tissue, allowing tumors to acquire traits that promote survival and malignancy. Given the importance of such interactions with respect to cancer susceptibility, development and progression, we need to better understand the molecular events and cellular contexts underlying epistasis. Knowledge of how environmental elements affect disease and how gene-gene and/or gene-environment interactions promote tumorigenesis, along with phenotypic categorization of tumor-associated hallmarks, has provided a framework for understanding how genetic factors contribute to oncogenesis [1, 2].

### Epistasis in disease susceptibility

Bateson coined the term ‘epistasis’ to explain interactions between two genes [3]. In this situation, the observable phenotype of one gene is masked by the other gene's effect, and the masking gene is said to be epistatic to the masked gene. Epistasis leads to deviation from simple Mendelian segregation ratios and emergence of novel phenotypes in a combination of single gene alleles. Since its first use, this term has been employed with diverse, and sometimes obscure, meaning.

In contrast to Bateson's perspective of biological epistasis, Fisher used quantitative genetics to explain the interaction between genetic loci that determine quantitative traits, rather than discrete binary traits within a group of genetically heterogeneous backgrounds [4]. Fisher's term, ‘epistacy,’ indicates a deviation from the addition of quantitative effects or phenotypes of two alleles in a given population.

In ‘statistical epistasis,’ the observed phenotype resulting from the interaction of two genes can be influenced both by allele frequency within a population and penetrance of the effects, which should still be visible after averaging multi-locus effects. Because the phenotype of interest, e.g. disease incidence, is arbitrarily determined by the investigator, genes in epistatic relationships can be discovered only when the effect of the combined genes is great enough to reach a penetrance threshold, resulting in a visible phenotype.

In this review, we investigate the interaction between two genes with statistically defined epistasis. We set the measure of individual function or effect of each allele as the disease susceptibility in a population, thus making it an odds ratio (OR). When the effects of particular alleles in two genes are OR_1_ and OR_2_, the phenotype observed with a combination of both alleles is represented as OR_combined_, while the epistatic deviation from the simple multiplication of two ORs for the individual gene alleles is represented as OR_interaction_ = OR_combined_ / (OR_1_ × OR_2_).

### Synergism and antagonism in epistasis

**Figure 1:**
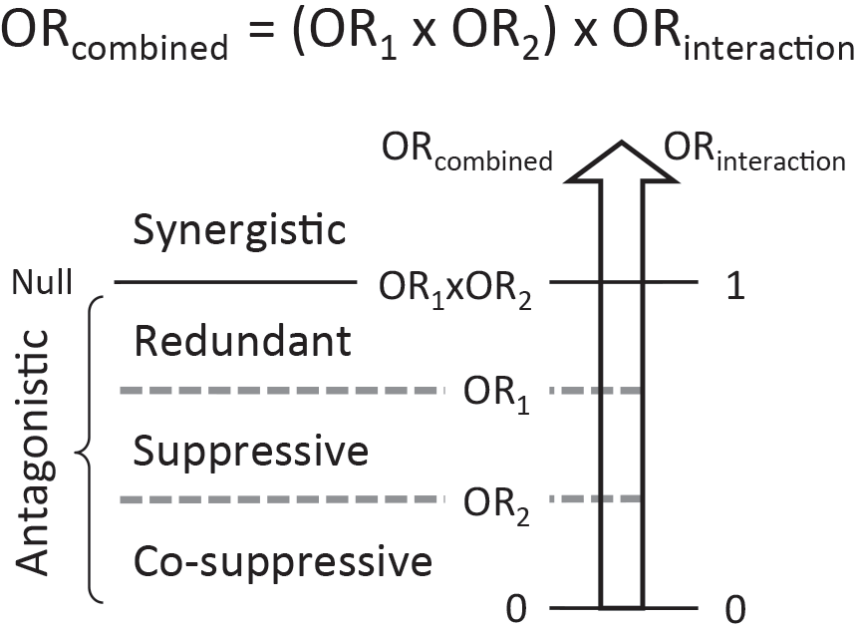
Quantitative levels of statistical epistasis Epistasis can be synergistic or antagonistic, if not null, and antagonistic epistasis can be redundant, suppressive or co-suppressive. OR represents the effect of each gene allele on disease susceptibility. OR_combined_ is OR observed for a combination of two gene alleles. OR_interaction_ is deviation of OR_combined_ from (OR_1_ × OR_2_). If OR_combined_ = (OR_1_ × OR_2_), or OR_interaction_ = 1, the interaction is ‘null,’ as the two individual gene allele effects are each additive to the other, with no interaction between them. Therefore, they act independently of each other. If OR_combined_ > (OR_1_×OR_2_), or OR_interaction_ > 1, the interaction is considered ‘synergistic,’ as the two individual alleles, when combined, act synergistically. Conversely, if OR_combined_ < (OR_1_×OR_2_), or OR_interaction_ < 1, two gene alleles have an ‘antagonistic’ effect. Antagonistic interactions can be further subcategorized into redundant, suppressive and co-suppressive interactions depending on the extent to which OR_combined_ departs from the two individual ORs, OR_1_ and OR_2_. If OR_combined_ ≥ OR_1_ ≥ OR_2_, the interaction is considered ‘redundant,’ as one gene allele effect is redundant with the other gene allele effect. If OR_1_ > OR_combined_ ≥ OR_2_, the interaction is considered ‘suppressive’ because one gene allele suppresses the other's effect. Lastly, if OR_combined_ ≤ OR_2_ < OR_1_, the interaction is ‘co-suppressive,’ as both gene alleles suppress each other.

Epistasis can be either ‘synergistic’ or ‘antagonistic,’ if not ‘null,’ (Figure 1) according to whether the outcome of the genetic interaction potentiates or diminishes the effect of the individual gene alleles [4–6]. Antagonistic interactions can be further divided into ‘redundant,’ ‘suppressive’ and ‘co-suppressive’ interactions depending on the extent to which OR_combined_ departs from the two individual ORs. Thus, levels of non-null epistasis can be described as synergistic, redundant, suppressive or co-suppressive (Figure 1).

To achieve synergism or antagonism, cooperation or a feedback loop is triggered when the combined effect of two interacting gene alleles surpasses a threshold necessary for activation of the feedback loop. In this feedback threshold model, a positive feedback loop potentiates the system and results in synergism, whereas a negative feedback loop diminishes the combined effect of the two alleles and leads to antagonism.

Synergism and antagonism may also be determined by qualitative regulation. When the interaction of two gene alleles, but not the individual activities of these alleles, results in a ‘gain of function’ with a novel ‘*Gestalt*’ product or activity that either enhances or suppresses the system, synergism or antagonism may ensue, respectively. The interaction scheme whereby two gene products operate in a linear pathway and the upstream gene allele effect is dominant over the downstream gene allele effect (i.e., if the observed OR_combined_ is the same as the OR of the dominant gene allele) is explained by a ‘dominance’ model in which the upstream gene allele effect masks the downstream gene allele effect.

### Review scope

We searched the PubMed, Google Scholar and Web of Science databases for studies associating statistical epistasis with susceptibility to gastrointestinal cancers, such as colorectal (colon, rectal or both), gastric (stomach) and esophageal (gullet) cancers. Sixteen studies met our selection criteria among those published before July 8, 2015. Search criteria and results are summarized in Table 1, where all 22 reported epistasis pairs are grouped into three categories: (i) interactions between cancer-associated genes, (ii) interactions between cancer-associated and non-associated genes and (iii) interactions between non-associated genes.

**Table 1:** Gene-gene interactions in gastrointestinal cancer susceptibility

Gene 1		Gene 2				Cancer	*n* (case/control)
Gene name (variation)^a^	OR^b^	Gene name (variation)^a^	OR^b^	OR_combined_^b^	OR_interaction_^c^	type^d^	ethnicity [reference]^e^
Interaction between cancer-associated gene variations							
*PARP1* (p.Val762**Ala**)	2.17	*XRCC1* (p.Arg399**Gln**)	1.61	6.43	1.84	GC	500/1000 Chinese [17]
*ADH1B* (p.**Arg**47His)	(3.34)	*ALDH2* (p.Glu504**Lys**)	(2.51)	13.46 (8.08)	1.60' (0.96)	EC	4220/8946 Asian [30]
*ADH1B* (p.**Arg**47His)	1.85	*ALDH2* (p.Glu504**Lys**)	1.66	6.79	2.21	EC	1070/2836 Japanese [29]
*MDM2* (rs2279744 *T*>***G***)	1.94	*TP53* (p.Arg72**Pro**)	1.72	5.05	1.51	GC	500/1000 Chinese [59]
*MUTYH* (p.Gln324**His**)	3.35	*OGG1* (p.Ser326**Cys**)	1.83	8.31	1.36	CRC	182/200 Polish [9]
*XRCC1* (p.Arg399**Gln**)	2.03	*OGG1* (p.Ser326**Cys**)	1.83	4.97	1.34	CRC	182/200 Polish [9]
*TP53* (p.Arg72**Pro**)	(1.78)	*MDM2* (rs2279744 *T*>***G***)	(1.46)	3.10	1.19'	EC	758/1420 Chinese [58]
*CHEK2* (**quadruple**)^f^	(1.38)	*CDKN1B* (p.**Val1**09Gly)	(1.34)	(2.14)	(1.16)	CC	872/3812 Polish [72]
*TPH2* (rs10879357 *G*>***A***)	(1.22)	*CASC20* (rs1571218 *G>****T***)	(1.20)	1.63 (1.55)	1.11' (1.06)	CRC	10907/13216 mixed [81]
*SMAD7* (rs11874392 *T*>***A***)	1.47	*TGFBR1* (rs6478972 ***A***>*G*)	1.38	*1.44* (1.50)	0.71 (0.74)	CRC	794/842 Chinese [69]
Interaction between cancer-associated and non-associated gene variations							
*CYP1A1* (p.Ile462**Val**)	(2.87)	*CYP2E1 (rs2031920* ***C****>T)*^g^	*(1.62)*	(11.25)	(1.85)	EC	526/526 Kashmir [48]
*PRKAG2* (rs1104897 *G*>***A***)	(1.30)	*RPS6KB1* (rs180515** ***A***>*G*)	(*1.05*)	(1.63)	(1.19)	RC	791/999 mixed^h^[73]
*NQO1* (p.Pro187**Ser**)	6.65	*NQO2* (rs2070999 *G*>***A***)	*1.48*	11.41	1.16	EC	135/195 Kashmiri [37]
*MUTYH* (p.Gln324**His**)	(4.05)	*XRCC1* (p.**Arg**194Trp)	*(1.24)*	(5.65)	(1.13)	CRC	182/200 Polish [9]
*GSTT1* (Δ)	(1.81)	*APEX1* (p.Asp148**Glu**)	(*1.21*)	1.84	0.84'	GC	314/548 Italian [23]
*PIK3CA* (rs7640662 ***C***>*G*)	(1.31)	*RPS6KB1* (rs180519 ***G***>*A*)	(*1.13*)	*(1.19)*	(0.80)	RC	791/999 mixed^h^ [73]
*GSTM1* (Δ) and *GSTT1* (Δ)	(2.50)	*APEX1* (p.Asp148**Glu**)	(*1.21*)	2.32	0.77'	GC	314/548 Italian [23]
*GSTM1* (Δ)	(2.24)	*NAT2* (p.Arg197**Gln**)	(1.81)	(2.82)	(0.70)	CRC	150/162 Romanian [45]
*PARP1* (p.Val762**Ala**)	(2.19)	*MMP2* (rs243865 *C*>***T***)	(*1.05*)	(*1.38)*	(0.60)	GC	59/320 Korean [79]
*OGG1* (p.Ser326**Cys**)	(1.71)	*XRCC1* (p.**Arg**194Trp)	(*1.22*)	(0.92)	(0.44)	CRC	182/200 Polish [9]
Interaction between non-associated gene variations							
*XPC* (PAT S>***L***)	(*1.20*)	*XPA* (rs1800975 *G*>***A***)	(*1.02*)	2.15 (*1.55*)	1.76' (1.27)	GC	314/548 Italian [23]
*MDM2* (rs2279744 *T*>***G***)	*(1.11)*	*TP53* (p.Arg72**Pro**)	*(1.06)*	1.78 (1.76)	1.52' (1.50)	CRC	444/569 Han Chinese [57]

a The interacting gene variant pairs are listed in descending order of OR_interaction_ value, shown in the sixth column. Nonsynonymous SNPs are denoted by the encoded amino acid changes, while the other variations are denoted by the reference SNP (rs) numbers. Risk-associated alleles that are more frequent in cancer cases than in healthy controls are shown in bold.

b Odds ratios (ORs) of the risk-associated alleles for cancer susceptibility are shown. ORs of non-significant associations are italicized. If the publication presented OR of non-risk allele rather than risk allele, crude OR of risk allele was calculated using the χ^2^ test and is shown here in parenthesis.

c OR_interaction_ = OR_combined_ ÷ (OR_1_ × OR_2_). When an adjusted OR_interaction_ value was not reported in the publication, OR'_interaction_ (marked with a prime) was calculated using the reported adjusted OR_combined_ and the crude OR_1_ and OR_2_ that were calculated using the reported genotype data. Alternatively, crude OR_interaction_ values were calculated using crude OR_combined_, OR_1_ and OR_2_ values and are parenthesized here.

d CC = colon cancer, CRC = colorectal cancer, EC = esophageal cancer, GC = gastric cancer, RC = rectal cancer.

e *Criteria for publication search and study selection.* This review included only studies where OR_interaction_ shown in the sixth column is >1.10 or <0.91. A search for articles in PubMed, Google Scholar and Web of Science, published through July 8, 2015, was conducted using the following keywords: ‘epistasis’ or ‘gene-gene interaction’ or ‘epistatic interaction’; and ‘esophageal cancer’ or ‘gastric cancer’ or ‘colorectal cancer’ or 'colon cancer' or 'rectal cancer' or ‘intestine cancer’.

f The quadruple variation of *CHEK2* includes del5395, 1100delC, c.444+1G>A and I157T [72].

g Only rs2031920 (*C*>*T*) of *CYP2E1*, perfectly correlated (*r*^2^ = 1) with rs3813867 (*G*>*C*), is shown here [48].

h The mixed population includes non-Hispanic Caucasians, Hispanics, American Indians, African Americans and Asians [73].

In principle, both synergistic and antagonistic interactions can be observed in each category, although underlying regulatory mechanisms may be different within distinct categories. In the cases where two variants interact, but are not individually associated with cancer risk, or where only one variant is associated with risk, effects are likely to be qualitative. In contrast, the interaction between two risk-associated variants may exert effects through both quantitative and qualitative mechanisms.

Here, we summarize previous studies demonstrating the role of epistasis in susceptibility to gastrointestinal cancers. Only cancers arising in the digestive tract, but not in accessary organs such as liver, are surveyed in this review. We also list and define the types of epistatic relationships between cancer susceptibility genes, and categorize the genes based on their functions and corresponding cancer hallmarks (Table 2).

**Table 2:** Predicted hallmarks and modes of biological interaction

Partner 1	Partner 2	Direct binding	Linear pathway	Convergence
*Genome instability hallmark*				
OGG1	XRCC1	Synergistic and Co-suppressive^a^		
PARP1	XRCC1	Synergistic		
XPA	XPC	Synergistic		
TP53	MDM2	Synergistic		
TGFBR1	SMAD7	Suppressive^b^		
MUTYH	OGG1		Synergistic	
MUTYH	XRCC1		Synergistic	
ADH1B	ALDH2		Synergistic	
NQO1	NQO2		Synergistic	
CYP1A1	CYP2E1			Synergistic
GSTT1	APEX1			Redundant
GSTT1/GSTM1	APEX1			Suppressive
GSTM1	NAT2			Redundant
*Proliferation hallmark*				
RPS6KB1	PRKAG2		Synergistic	
RPS6KB1	PIK3CA		Suppressive	
CDKN1B	CHEK2			Synergistic
*Multiple hallmarks*^c^				
MMP2	PARP1			Suppressive

a An *OGG1* SNP showed a synergistic interaction with p.Arg399Gln SNP (rs25487) but a co-suppressive interaction with p.Arg194Trp SNP (rs1799782) of *XRCC1* [9].

b The interaction was suppressive with the adjusted OR_combined_, but redundant with the crude OR_combined_ [69].

c Multiple hallmarks include inflammation, proliferation, insensitivity, resistance, immortality and angiogenesis for MMP2, and mutation for PARP1.

## EPISTASIS OF GENOME INSTABILITY AND MUTATION

The ten current hallmarks of cancer include two cancer-enabling characteristics and eight cancer-associated features [2]. One enabling characteristic is genome instability and mutation. DNA can be damaged by genotoxic stresses from environmental agents, such as UV, ionizing radiation and DNA-altering chemicals. Endogenous agents that originate from normal cellular metabolism, such as reactive oxygen species, can also cause DNA damage. Defects in genome maintenance and DNA repair are observed in numerous tumors and may lead to genetic errors and tumorigenesis.

### Base excision repair

The base excision repair system recognizes small DNA lesions in the form of single-strand nicks or aberrant bases modified by oxidation, reduction or methylation. Damaged bases are first removed by DNA glycosylases. 8-Oxoguanine (8-oxoG), an oxidized derivative of guanine (G) made by reactive oxygen species, can pair with adenine (A) rather than cytosine (C) during replication, leading to G:C to T:A conversion if not repaired. This A is excised by adenine glycosylase MUTYH and replaced with C in subsequent base excision repair reactions. The resulting 8-oxoG:C pair is then excised by glycosylase OGG1 [7].

The rs1052134 single-nucleotide polymorphism (SNP) in the *OGG1* gene encodes p.Ser326Cys, a substitution of Ser for Cys at amino acid 326 of the OGG1 protein. This *OGG1* SNP interacts synergistically with *MUTYH* SNP rs3219489, encoding p.Gln324His and affecting glycosylase activity, to increase colorectal cancer risk (OR_interaction_ = 1.36) [8, 9]. The OGG1 Cys variant had multiple defects in DNA binding, repair and/or nuclear localization [10–12]. This *OGG1*-*MUTYH* interaction demonstrates the possibility that two genes within the same pathway can be mutually influential in altering cancer susceptibility, although their gene products do not necessarily physically interact with each other.

OGG1 directly interacts with XRCC1 [13, 14], and the *OGG1* rs1052134 showed epistasis with two *XRCC1* SNPs in colorectal cancer risk [9]. These included a synergistic interaction (OR_interaction_ = 1.34) with *XRCC1* rs25487 encoding p.Arg399Gln, and a co-suppressive interaction (crude OR_interaction_ = 0.44) with *XRCC1* rs1799782 encoding p.Arg194Trp. (Whereas OR_interaction_ values were calculated using adjusted OR_1_, OR_2_ and OR_combined_ values for the risk-associated alleles, ‘crude OR_interaction_’ values were calculated using crude OR_1_, OR_2_ and OR_combined_ values from the reported data when their adjusted values for the risk-associated alleles were not available. Crude OR values are parenthesized in Table 1.) Incidentally, the *XRCC1* rs1799782 synergistically interacted (crude OR_interaction_ = 1.13) with the *MUTYH* rs3219489, albeit weakly, in colorectal cancer risk [9].

PARP1 is activated following sensing of nicked DNA and is auto-ribosylated. It recruits XRCC1-ligase 3 to regions of single-strand nicks, where the ribosyltransferase activity of PARP1 is required. PARP1 directly binds XRCC1 at a central region containing the BRCT domain [15, 16]. Susceptibility to gastric cancer is associated with *PARP1* rs1136410 encoding p.Val762Ala [17], along with the Ala variant, which displayed diminished enzymatic activity [18]. The *PARP1* rs1136410 synergistically interacted (OR_interaction_ = 1.84) with *XRCC1* rs25487 encoding p.Arg399Gln in gastric cancer risk [17]. Both risk-associated PARP1 and XRCC1 substitutions are expected to decrease base excision repair through reduced PARP1 ribosylation and XRCC1 recruitment. Because PARP1 can also induce cell death by enhancing nuclear translocation of apoptosis-inducing factor, PARP1 modifications merit further investigation.

**Figure 2:**
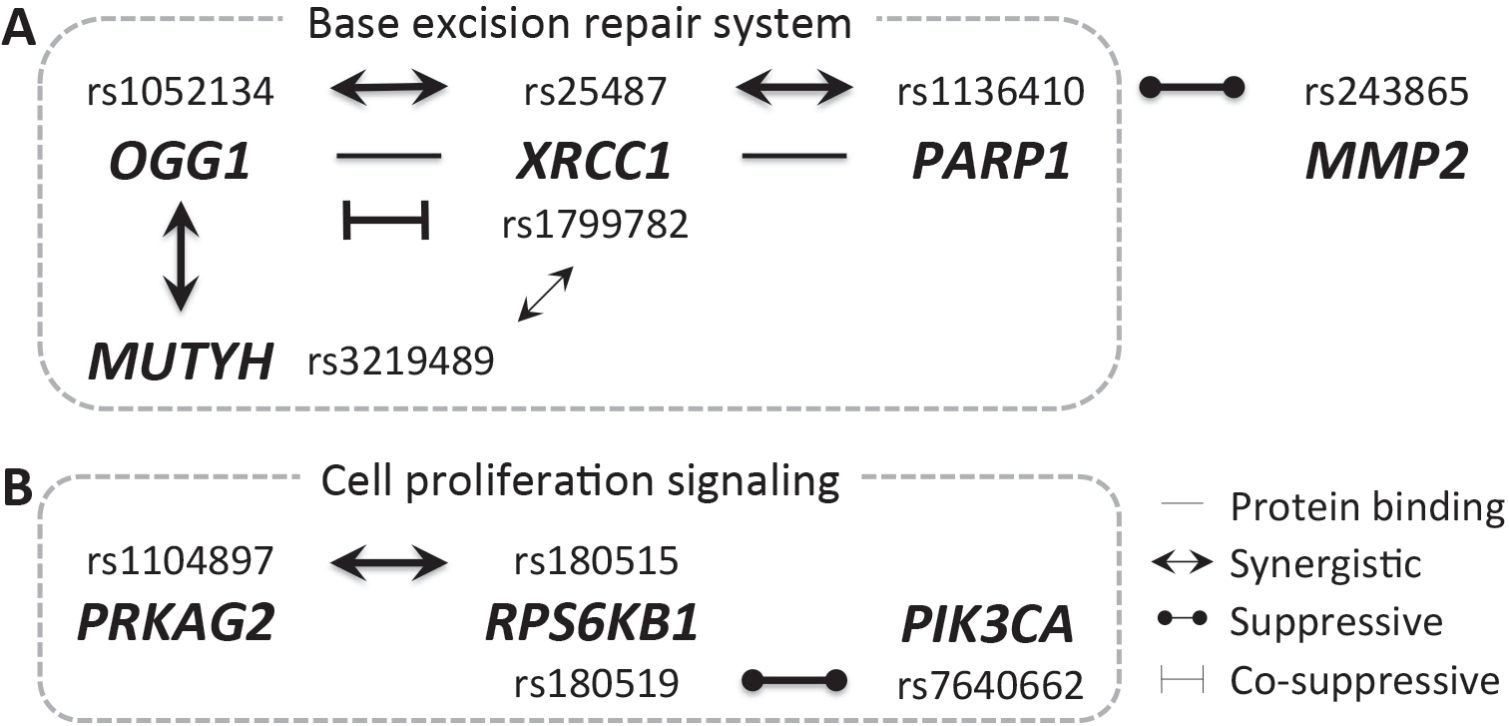
Topology of epistasis networks **A.** Five genes, primarily part of the base excision repair system, form a linear or lariat epistasis network. **B.** Three genes involved in mTOR signaling of cell proliferation form a linear epistasis network.

The above findings demonstrate multiple epistatic relationships between *MUTYH*, *OGG1*, *XRCC1* and *PARP1* of the base excision repair system. These interactions, excluding the weak interaction between *MUTYH* and *XRCC1* (rs1799782), may shape a linear network topology in the form of *MUTYH*-*OGG1*-*XRCC1*-*PARP1* (Figure 2A), although the three interaction links were observed in two different studies for different cancers (two links in Polish colorectal cancer [9] and one in Chinese gastric cancer [17]). Every direct link in this linear network was synergistic. Therefore, super-synergism can be expected for carriage of all risk-associated alleles in these four genes.

### Nucleotide excision repair

The nucleotide excision repair system recognizes bulky DNA lesions, including UV-induced pyrimidine dimers, photoadducts and chemical adducts. Damaged DNA is recognized by a complex of XPA, XPC and RPA [19]. The SNP, rs1800975, in the 5′ untranslated region of *XPA*, just four nucleotides upstream of the AUG start codon, is part of the Kozak sequence, which affects translation initiation efficiency. The major allele *G* was associated with a higher DNA repair capacity than the minor allele *A* in Caucasians [20–22].

This *XPA* rs1800975 synergistically interacted (OR’_interaction_ = 1.76) with an *XPC* polymorphism, PAT S>L, in gastric cancer risk [23]. (Here, an OR’_interaction_ value, marked with a prime symbol, was calculated using a reported adjusted OR_combined_ value and the crude OR_1_ and OR_2_ values that we calculated using the reported genotype data.) The *XPC* PAT polymorphism alone was not associated with cancer risk, but enhanced the effect of *XPA* rs1800975. The *XPC* PAT polymorphism is in linkage disequilibrium with *XPC* rs2228001 encoding p.Lys939Gln. This nonsynonymous SNP did not affect the XPC function *in vitro* [24], and the effect of the PAT polymorphism here remains to be determined.

### Detoxification in alcohol metabolism

Alcohol consumption is an environmental risk factor for certain cancers. Alcohol is metabolized into acetate through two steps. First, ethanol is oxidized to acetaldehyde by alcohol dehydrogenase, primarily ADH1B. Then, acetaldehyde is further oxidized to acetate by acetaldehyde dehydrogenase ALDH2. With respect to *ADH1B* rs1229984 encoding p.Arg47His, the Arg allele enzyme has lower catalytic activity for ethanol metabolism than the His allele enzyme [25, 26]. Regarding *ALDH2* rs671 encoding p.Glu504Lys (also known as Glu487Lys), the Lys allele enzyme has lower activity than the Glu allele enzyme [27].

SNPs in both *ADH1B* and *ALDH2* are associated with susceptibility to esophageal and gastric cancers in alcohol drinkers [28]. Synergistic interaction between *ADH1B* and *ALDH2* SNPs was observed in a study of Japanese esophageal cancer (OR_interaction_ = 2.21) [29], and also in a subsequent meta-analysis (OR’_interaction_ = 1.60) [30]. Further study is required to determine how the interaction between the two lower activity ADH1B (47Arg) and ALDH2 (504Lys) variants leads to increased cancer risk. One interesting possibility is that un-metabolized ethanol reaches the intestinal mucosa, where it is converted to harmful acetaldehyde by gut microflora and thereby increases colon cancer risk [31].

### Detoxification in quinone metabolism

Another example of environmental factors in cancer risk is NQO1 and NQO2 of quinone metabolism. Carcinogenic quinones and polycyclic aromatic hydrocarbons are detoxified by these flavoprotein enzymes, which thereby protect cells from redox cycling and oxidative stress. Polycyclic aromatic hydrocarbons are contained in exogenous chemicals such as tobacco smoke, automobile exhaust and burnt foods.

Several studies have associated *NQO1* polymorphisms with susceptibility to esophageal and cutaneous (skin) cancers [32, 33]. The Ser variant of *NQO1* rs1800566 encoding p.Pro187Ser has reduced enzymatic activity [34, 35] and is associated with higher benzene toxicity compared with the Pro variant [36]. This *NQO1* SNP was associated with esophageal adenocarcinoma risk and synergistically interacted (OR_interaction_ = 1.16) with a promoter SNP of *NQO2*, rs2070999, which was not associated with esophageal adenocarcinoma risk [37]. The functional role of this *NQO2* SNP in gastrointestinal cancer remains to be elucidated.

### Detoxification by glutathione conjugation

Glutathione S-transferases (GSTs) form a large family of detoxifying enzymes that neutralize electrophiles and radicals by conjugating environmental carcinogens to reduced glutathione. Within the GST family genes, *GSTT1* and *GSTM1* are frequently deleted (referred to as the null genotype) in the general population. Carriers of the null genotype were more susceptible to cancer than non-carriers [38].

A case-control study separately associated *GSTT1*- and *GSTM1*-null genotypes with increased gastric cancer risk [39]. Carriers of the double-null genotype, lacking both *GSTT1* and *GSTM1*, display increased gastric cancer risk relative to non-carriers in an Italian population and by a meta-analysis [40, 41]. Deletion of GSTs might be associated with high levels of *Helicobacter pylori*-induced reactive oxygen species and thus increase susceptibility to inflammation-related cancer through enhanced DNA damage.

*APEX1* rs1130409 encoding p.Asp148Glu showed redundant interaction (OR’_interaction_ = 0.85) with the *GSTT1-*null genotype, but suppressive interaction (OR’_interaction_ = 0.77) with the *GSTT1-GSTM1* double-null genotype in gastric cancer susceptibility [23]. APEX1 is a key enzyme involved in base excision repair, which regulates chemosensitivity. *APEX1* expression is increased with *H. pylori* infection, which plays a critical role in gastric cancer development [42]. However, this nonsynonymous *APEX1* SNP did not alter protein function or structure [43].

Alone, *APEX1* rs1130409 was not associated with gastric cancer risk, suggesting that this SNP has an impact only in conjunction with the *GSTT1*- or double-null genotype. Given that GSTs protect against DNA damage and that APEX1 participates in DNA repair, cooperationbetween DNA damage prevention and repair appears critical for maintenance of genome integrity and underlies inter-individual variability in gastric cancer risk.

The *GSTM1* null genotype was associated with higher risk for colorectal and gastric cancers [7, 44]. Additionally, this null genotype redundantly interacted (crude OR_interaction_ = 0.70) in sporadic colorectal cancer risk with *NAT2* rs1799930 encoding p.Arg197Gln [45]. NAT2 mediates acetylation of aromatic and heterocyclic carcinogenic amines [46], which can detoxify or activate the chemicals.

Pro-carcinogens present in tobacco, such as nitrosamines and polycyclic aromatic hydrocarbons, are metabolized into active carcinogens by cytochrome P450 proteins (CYPs). For example, CYP1A1, also known as aryl hydrocarbon hydroxylase, catalyzes metabolic activation of benzo(a)pyrene into a carcinogenic epoxide [47]. Synergistic interaction between *CYP1A1* rs1048943 encoding p.Ile462Val and *CYP2E1* rs2031920 was observed in Kashmir esophageal cancer (crude OR_interaction_ = 1.85) [48].

### TP53 tumor suppressor pathway

Transcription factor TP53 is involved in DNA repair, cell-cycle arrest, senescence and apoptosis in response to various cellular stresses. Somatic, attenuating *TP53* mutations are found in many human cancers [49]. However, tumors can also arise from mutations in genes regulating the TP53 pathway without *TP53* mutation itself [50]. For example, expression of *MDM2*, encoding an E3 ubiquitin ligase, is elevated in various human tumors, and MDM2 negatively regulates TP53 transcriptional activity either directly or indirectly [51].

*TP53* and *MDM2* polymorphisms have been studied in regards to both susceptibility to cancer and responsiveness to cancer therapy. The Pro variant of *TP53* rs1042522 encoding p.Arg72Pro is less potent in inducing apoptosis than the Arg variant [52, 53]. However, the Pro allele confers higher apoptotic capacity during chemotherapy in the presence of some somatic tumor-associated *TP53* mutations, suggesting that the success of chemotherapy in inducing TP53-mediated apoptosis is dependent on a given patient's combined somatic and germline *TP53* modifications [54].

For *MDM2* rs2279744, the minor allele *G* has higher affinity for transcription factor SP1 than the major allele *T*, increases *MDM2* expression and attenuates TP53-mediated apoptosis [55]. Higher *MDM2* expression and consequent TP53 pathway attenuation are associated with susceptibility to lung and esophageal cancers [56, 57]. While MDM2 and TP53 function within the same pathway, synergistic interactions were found between the minor alleles of *MDM2* rs2279744 and *TP53* rs1042522 in increasing the risk of colorectal (OR’_interaction_ = 1.52), gastric (OR_interaction_ = 1.51) and esophageal (OR’_interaction_ = 1.19) cancers [57–59].

### Transforming growth factor pathway

Transforming growth factor TGF-β is multifunctional. It inhibits the cell cycle by suppressing c-Myc expression and enhancing expression of cyclin-dependent kinase inhibitors such as CDKN2B and CDKN1A [60]. Similar to TP53, TGF-β has both intrinsic effects on genomic stability, cell differentiation, senescence and apoptosis, and extrinsic effects on suppression of inflammation and mitogens.

Contrary to the tumor-suppressive role of TGF-β at early tumor development stages, malignant cells at late stages downregulate expression of TGF-β receptors and become resistant to TGF-β-mediated growth inhibition. Moreover, TGF-β signaling activation at late stages paradoxically promotes cancer cell metastasis by activating the epithelial-to-mesenchymal transition, modulating microenvironments and suppressing anti-tumor immune responses [61]. Therefore, the consequence of TGF-β signaling activation varies depending on cell type and context.

TGF-β signaling is initiated by its binding to a heterodimeric complex of type-I and type-II TGF-β receptors, TGFBRI and TGFBRII. This signaling is negatively regulated by SMAD7, which competes with R-SMADs for receptor binding and mediates degradation of the receptors *via* ubiquitination [62, 63]. Some TGF-β signaling genes were associated with colorectal cancer risk [64–68], and specifically *TGFBR1* rs6478972 and *SMAD7* rs11874392 exhibited suppressive epistasis (OR_interaction_ = 0.71) [69].

*TGFBR1* rs6478972 is in linkage disequilibrium with rs334348, which is located in the 3′ untranslated region and might affect microRNA binding and consequently TGFBR1** protein levels [70, 71]. Accordingly, the two interacting SNPs could affect expression of the two cancer-associated proteins, TGFBR1 and SMAD7, participating in the same TGF-β signaling pathway, but affecting multiple cancer hallmarks.

## EPISTASIS IN SUSTAINING PROLIFERATIVE SIGNALING

The next cancer hallmarks exhibiting epistasis are sustaining growth signals and insensitivity to anti-growth signals. These characteristics are related to the autonomous capability of cancer to increase cell numbers. The *CDKN1B* and *CHEK2* pair showed epistasis in colorectal cancer susceptibility. Among the four *CHEK2* polymorphisms associated with colon cancer risk, three variants (del5395, 1100delC and c.444+1G>A) produce truncated proteins, and one (I157T) is a missense mutant. These four *CHEK2* variations synergistically interacted (crude OR_interaction_ = 1.16) with *CDKN1B* rs2066827 encoding p.Val109Gly in colon cancer risk [72].

Cyclin-dependent kinases (CDKs) drive cell cycle progression, which is counter-regulated by CDK inhibitors. Pauses at the G_1_-S cell cycle checkpoint are mediated by CDKN1B, a cell cycle inhibitor, and CHEK2, a checkpoint protein activated by DNA damage and replication inhibition. CDKN1B inhibits the CDK2-cyclin E complex, preventing cells from progressing into S phase. CHEK2 functions, such as phosphorylating CDC25C phosphatase and stabilizing TP53, converge in inhibition of the CDK2-cyclin E and CDK4-cyclin D complexes. Therefore, CDKN1B and CHEK2 have different targets, but the same effect, namely, regulation of the G_1_-S checkpoint.

Additionally, *RPS6KB1* rs180515 exhibited synergistic epistasis (crude OR_interaction_ = 1.19) with *PRKAG2* rs1104897, but *RPS6KB1* rs180519 showed suppressive epistasis (crude OR_interaction_ = 0.80) with *PIK3CA* rs7640662 in rectal cancer susceptibility [73]. Although two different *RPS6KB1* SNPs are involved, *PRKAG2* may interact with *PIK3CA* indirectly through *RPS6KB1*, forming the linear interaction network, *PRKAG2*-*RPS6KB1*-*PIK3CA* (Figure 2B).

RPS6KB has two isoforms, RPS6KB1 and RPS6KB2, and is involved in protein synthesis necessary for cell growth in response to various growth factors, insulin and nutrients. RPS6KB is phosphorylated by the mTOR complex, which serves as a regulatory axis for cell growth and proliferation [74]. The mTOR pathway and RPS6KB are activated by PIK3CA and inhibited by PRKAG2 [75]. This sharing of a common regulatory target explains the linear interaction network, *PRKAG2*-*RPS6KB1*-*PIK3CA*.

## EPISTASIS OF OTHER COMPLEX HALLMARKS

### Epistasis between different cancer hallmark pathways

For tumor cell invasion and metastasis, the extracellular matrix and basement membrane must be degraded, and this in turn facilitates angiogenesis. The altered tissue environment around tumor cells is called the tumor microenvironment, and matrix metalloproteinases (MMPs) are crucial for shaping this microenvironment and promoting tumor progression [76].

Specifically, *MMP2* expression is associated with gastric cancer progression and lymph node metastasis [77]. An *MMP2* promoter SNP, rs243865, affects gene expression by altering SP1 transcription factor binding [78]. This functional SNP was not associated with gastric cancer susceptibility, but its minor allele suppressed the gastric cancer risk-enhancing effect of the minor allele in the above-mentioned *PARP1* rs1136410 (crude OR_interaction_ = 0.60) [79].

The interaction between *PARP1* and *MMP2* would extend the *MUTYH*-*OGG1*-*XRCC1*-*PARP1* linear interaction network to *MUTYH*-*OGG1*-*XRCC1*-*PARP1*-*MMP2* (Figure 2A). However, the *PARP1*-*MMP2* link in this network is suppressive, unlike the other three synergistic interactions, and has been observed in different studies (Korean gastric cancer [79] *versus* Chinese gastric cancer [17] and Polish colorectal cancer [9]).

PARP1 participates in the base excision repair system, but not in any pathway known to involve MMP2. This is the only case reported to date where two statistically interacting genes are involved in apparently distinct pathways, but crosstalk has not been detected. If the statistical epistasis between *PARP1* and *MMP2* is related to cancer risk, there must be crosstalk or merging of the two pathways, incorporating disparate cancer traits. Although multiple MMP-conferred effects add complexity to the understanding of epistasis in cancer, dissecting this link at the molecular level could be relevant for improved therapeutics.

### Epistasis of noncoding RNA

Lastly, there was a weakly synergistic interaction (OR’_interaction_ = 1.11) between intronic rs10879357 of *TPH2* and intergenic rs1571218 at human chromosome 20p12.3 in colorectal cancer susceptibility [81]. Because both rs1571218 and nearby rs961253 are associated with colorectal cancer risk [81, 82], this locus cancer risk association appears to be replicated. These two intergenic SNPs are both closer to the long noncoding RNA gene *CASC20* than to nearby *BMP2*, but no function is yet known for CASC20.

Meanwhile, TPH2 participates in serotonin synthesis and lack of serotonin has been associated with tumor growth reductions in a mouse model of colon cancer allografts [83]. It will be worthwhile to determine whether CASC20 regulates *TPH2*, because many long noncoding RNAs regulate diverse gene expression steps [84].

## MODE OF BIOLOGICAL INTERACTION FOR STATISTICAL EPISTASIS

**Figure 3:**
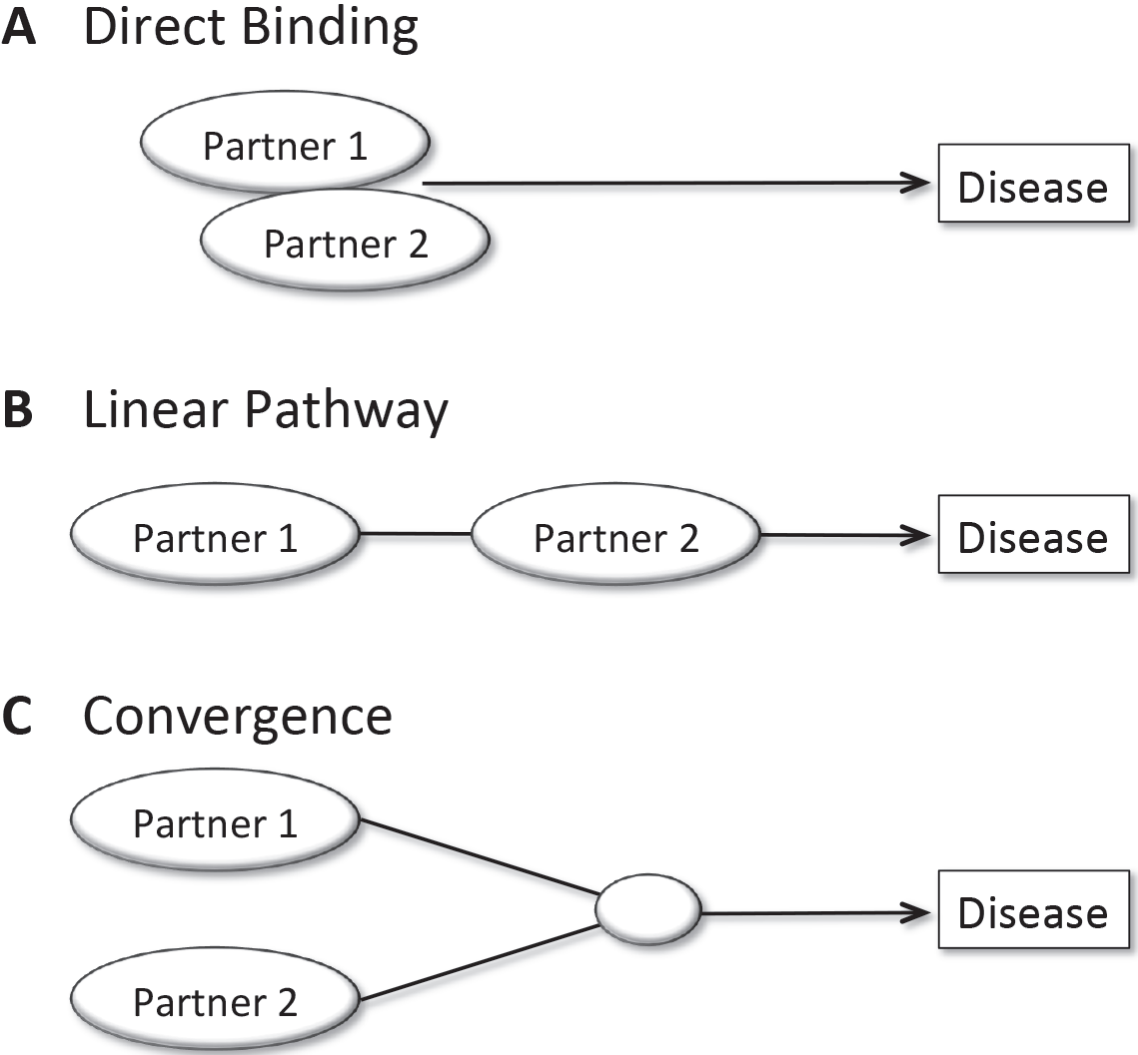
Three plausible modes of biological interaction between two statistically epistatic gene products **A.** In the direct binding mode, two partners physically bind to each other and function together to affect disease susceptibility. **B.** In the linear pathway mode, two partners work sequentially. **C.** In the convergence mode, two partner-involving pathways converge to promote disease susceptibility.

All four levels of epistasis, i.e. synergistic, redundant, suppressive and co-suppressive (Figure 1), have been observed for susceptibility to gastrointestinal cancers (Table 2). We propose here that all statistical epistases can be explained by three different modes of biological interaction: ‘direct binding,’ ‘linear pathway’ and ‘convergence’ modes (Figure 3).

In the direct binding mode, two gene products (proteins or noncoding RNAs) physically bind to each other and the final combined effect is determined by combinatorial codes. Therefore, their interaction would be expected to be synergistic or antagonistic in altering disease susceptibility. This principle is exemplified by the five direct-binding pairs, where one partner is already known to directly control the other; OGG1-XRCC1, XRCC1-PARP1, XPA-XPC, TP53-MDM2 and TGFBR1-SMAD7 (Table 2)

Without direct binding between their products, two genes could still be epistatic by participating in the same pathway (linear pathway mode) or two distinct, but converging pathways (convergence mode). When one partner precedes the other in a linear pathway leading to cancer development, their cooperation increases cancer susceptibility. For example, OGG1-MUTYH-XRCC1 in base excision repair, ADH1B-ALDH2 in alcohol metabolism, NQO1-NQO2 in quinone metabolism and PRKAG2-RPS6KB1-PIK3CA in the mTOR pathway all exhibit the linear pathway mode of interaction (Table 2).

Convergence mode, where two pathways merge and proceed together in a single pathway, is also observed (Table 2). CDKN1B and CHEK2 each have distinct target proteins, but both eventually participate in the G_1_-S checkpoint, regulating the cell cycle. Both the GSTT1 and APEX1 pathways act in DNA repair, and GSTM1 and NAT2 promote detoxification. Notably, PARP1 and MMP2 are not known to participate in a single linear or converged pathway, but their interaction suggests as-yet unidentified crosstalk between their respective pathways.

## EMERGING PERSPECTIVES ON CANCER GENETICS

Several new perspectives have surfaced from the last decade of cancer genetics research. First, some genes originally classified as part of a core cancer hallmark in fact exert pleiotropic effects on multiple hallmarks. For example, TP53 functions as a growth suppressor, a mediator for senescence and apoptosis, and a guardian of the genome [10].** TGF-β has either tumor-suppressing or tumor-promoting activities, depending on the context [14].

Second, crosstalk between different cancer hallmarks is increasingly explored. Epistasis in cancer susceptibilityis detected more frequently and with increasing statistical power. Some epistases support known biological crosstalk, but others suggest previously unidentified crosstalk, as exemplified by the *PARP1*-*MMP2* interaction [79]. Dissection of crosstalk mechanisms among interacting gene pathways merits further investigation, and will provide a deeper understanding of cancer susceptibility.

Third, complex interaction network topological structures are expected, as point-to-point pairwise interactions are increasingly observed in disease susceptibility studies. For example, in this review, a linear topology is inferred for the interaction network, *MUTYH*-*OGG1*-*XRCC1*-*PARP1*-*MMP2* (Figure 2A), although not all links were observed in the same cancer for a single ethnic population. Therefore, this network must be verified using the same study population for the same disease, incorporating a large enough number of samples to achieve sufficient statistical power. The topology does not need to be linear as in this example. Indeed, even this example could become a ring, mesh or other structure upon discovery of additional interaction links.

Fourth, epigenetic dysregulation is an emerging cancer characteristic. Comprehensive mapping of epigenetic landscapes and noncoding RNAs in cancer cells has revealed that they play essential roles in cell proliferation, apoptosis and metastasis [85]. Noncoding RNAs and epigenetic signatures representing DNA methylation, histone modification and nucleosome remodeling in gastric and colorectal cancers have been compiled [86–89] and applied to improve diagnoses, prognoses and therapeutic interventions [90, 91]. Epigenetic polymorphisms in cancers, as well as interactions between epigenetic and genetic polymorphisms in cancer susceptibility and hallmark genes, must be further investigated to identify meaningful epistatic interactions.

Fifth, multiple roles for a particular gene in various cancer traits can be clearly elucidated if the context in which the gene exerts its effect is understood. In tumor microenvironments, neoplastic cells interact with not only stromal cells, but also immune cells infiltrating tumor tissues, inducing an inflammatory response. Inflammation in the microenvironment can promote tumor cell proliferation, survival, vascularization, invasion, metastasis and epithelial-to-mesenchymal transition [11].

Finally, heterogeneity of somatic cells in tumor tissues is evident in whole-genome sequencing of individual tumor cells. Thus, single-cell analyses can reveal whether cancer susceptibility genes are mutated in an individual tumor cell, whether any germline polymorphisms are associated with somatic mutations of cancer hallmark genes, and whether there is epistasis between germline polymorphisms and somatic mutations in promotion of tumor development and progression.

## CONCLUDING SUMMARY

In this review, we surveyed statistical gene-gene interactions reported for genetic susceptibility to colorectal, gastric and esophageal cancers (Table 1). We found multiple gene pairs participating in synergistic, redundant, suppressive or co-suppressive interactions (Table 2), as statistical epitasis is defined according to the degree and direction of interaction between genetic variants in disease susceptibility (Figure 1). We inferred that more than two genes could indirectly interact with one another in cancer susceptibility to form a topological network structure (Figure 2).

We also proposed three different modes of biological interaction as the underlying molecular mechanisms for statistical epistasis. The direct binding, linear pathway and convergence modes can exhibit any level of statistical epistasis in disease susceptibility (Figure 3). Finally, several perspectives are provided here regarding interactions among germline polymorphisms, epigenetic variations and somatic mutations in cancer susceptibility.
